# The Elimination of Viroids through In Vitro Thermotherapy and a Meristem Tip Culture from a New Limonime Hybrid (*Citrus* x *limon* var. *limon* (L.) Burm. f. x *Citrus latifolia* var. *latifolia*)

**DOI:** 10.3390/biotech13030037

**Published:** 2024-09-23

**Authors:** Virginia Sarropoulou, Katerina Grigoriadou, Varvara I. Maliogka, Chrysoula-Lito Sassalou, Vasileios Ziogas

**Affiliations:** 1 Hellenic Agricultural Organization—DIMITRA (ELGO-DIMITRA), Institute of Plant Breeding and Genetic Resources, Thermi, 57001 Thessaloniki, Greece; vsarrop@gmail.com (V.S.); katgrigoriadou@elgo.gr (K.G.); 2 Laboratory of Plant Pathology, Faculty of Agriculture, School of Agriculture, Aristotle University of Thessaloniki, Forestry and Natural Environment, 54124 Thessaloniki, Greece; vmaliogk@agro.auth.gr (V.I.M.); sassalou@agro.auth.gr (C.-L.S.); 3 Hellenic Agricultural Organization—DIMITRA (ELGO-DIMITRA), Institute of Olive Tree, Subtropical Crops and Viticulture, 73134 Chania, Greece

**Keywords:** auxins, citrus exocortis viroid (CEVd), cytokinins, hop stunt viroid (HSVd), Zambetaki lemon x Tahiti lime, micropropagation, plant material sanitization, RT-PCR

## Abstract

Viruses and viroids pose a significant challenge in citriculture, and their control is crucial for plant health. This study evaluated the effectiveness of in vitro thermotherapy combined with a meristem tip culture for eliminating citrus exocortis viroid (CEVd) and hop stunt viroid (HSVd) from a new limonime hybrid (*Citrus* x *limon* var. *limon* x *Citrus latifolia* var. *latifolia*). The elimination success was confirmed by RT-PCR assays. The in vitro elimination rate for CEVd during the shoot proliferation stage (43%) was higher than for HSVd (21%). Accordingly, in the subsequent rooting stage, the in vitro elimination rate for CEVd (50%) was higher than for HSVd (33%). Successful CEVd and HSVd eradication at a 100% rate was confirmed in the ex vitro acclimatized plants in the greenhouse. The study also established an efficient micropropagation protocol. The optimal treatment for in vitro shoot induction was 0.5–2 mg L^−1^ benzyladenine (BA) + 0.5 mg L^−1^ gibberellic acid (GA_3_) + 0.25 mg L^−1^ naphthalene acetic acid (NAA), while for shoot elongation, it was 0.5 mg L^−1^ BA + 0.5 mg L^−1^ kinetin (KIN) + 0.5 mg L^−1^ GA_3_ + 0.25 mg L^−1^ NAA. Rooting was best promoted by 1 mg L^−1^ NAA. This study provides valuable insights for the mass production of viroid-free propagation material in this new lemon x lime hybrid, contributing to the conservation of genetic resources in citrus breeding programs through the combined application of in vitro thermotherapy and an in vitro meristem tip culture, a novel and highlighted achievement reported for the first time in this study.

## 1. Introduction

Citruses, a wide genus of fruit species consumed by humans with economic and medicinal value, are rich in vitamins mainly of ascorbic acid, minerals, and phytochemicals of strong antioxidant activity including essential oils, phenols, and flavonoids [1]. The fruits of *Citrus* species are sold and consumed either fresh or after undergoing processing in the form of juices, juice concentrate, jams, compotes, liqueurs, oils, and other products [2]. The International Treaty on Plant Genetic Resources recognizes citruses as crucial for crop diversity [3]. This diversity is necessary for use in breeding programs that seek to improve overall plant performance against biotic and abiotic challenges as well as to improve product quality to benefit the wellbeing and health of consumers [2]. The conservation of citrus trees and their wild crop relatives is globally supported by the Biodiversity International and the Citrus Germplasm Network [3] in the form of collections as plantings in the field or as potted trees in greenhouses or screenhouses [2]. Fungi, bacteria, viruses, viroids, phytoplasmata, and arthropods are threatening the genebank collections and commercial production of citruses. Accordingly, plant quarantine and sanitation programs are utilized at national and regional levels to confine the spread of these pathogens and pests [2]. The use of genetic resources through breeding for the development of new varieties that perform better adaptation or tolerance to these challenges constitutes a significant conservation strategy [2].

In the framework of the aforementioned information, the Institute of Olive Tree, Subtropical Plants, and Viticulture, ELGO–DIMITRA, Greece, has developed a new limonime (lemon x lime) hybrid, “*Citrus* x *limon* var. *limon* (L.) Burm. f. x *Citrus latifolia* var. *latifolia*”, through a Greek Ministry-funded research program. This particular hybrid is a new species that was included in the special program for the creation of new varieties with the Ministry of Rural Development and Food in Greece to be utilized and registered in the national list of varieties of plant species. This hybrid was the outcome of the crossing of Zambetaki lemon (*Citrus* x *limon* cv. *Zambetaki*) (father—provided the pollen) and Tahiti lime (*Citrus latifolia*) (mother—provided the pistil); thus, for this reason, both citrus species are included in its scientific name. Its creation, carried out by the process of classical genetic breeding (pollen collection and the pollination of flowers), was the successful result of multiple repeated attempts. Currently, it is kept in the facilities of ELGO–DIMITRA at Chania of Crete, S. Greece, and is evaluated for its arboricultural and quality characteristics.

Citruses face increasing phytosanitary challenges from pathogens (bacteria, viruses, fungi, phytoplasmas) threatening the industry globally [4]. In Greece, despite being a top fruit exporter, the citrus sector is in crisis due to competition, changing market trends, and diseases, especially those induced by viruses and viroids [5]. Viroids, small circular RNA pathogens, are spread through horticulture tools, grafting, pruning, and infected cuttings, posing a significant threat to citriculture [6]. Viruses and viroids severely impact citrus vigor, yield, and quality, leading to the exclusion of some cultivars from commercial use [7]. The impact of viroids on citrus crops depends on the species, variety, genotype, and environmental conditions [8].

Citrus exocortis viroid (CEVd) (genus *Pospiviroid*, family *Pospiviroidae*) and hop stunt viroid (HSVd) (genus *Hostuviroid,* family *Pospiviroidae*) have been reported in most Mediterranean countries and are among the most prevalent citrus viroids in the region [9] that cause destructive diseases like exocortis and cachexia-xyloporosis in citruses, resulting in yield losses of up to 34–76%, depending on viroid–rootstock–scion combinations [10,11]. CEVd induces stunting, bark issues, leaf abnormalities, petiole cracks, low fruit-bearing, and poor tree performance [12,13,14], while HSVd leads to gumming, phloem tissue browning, wood pitting, dwarfing, the reduction of growth and fruit yield, and the decline and death of severely affected trees in various citrus species [12,15,16]. In viroid/viroid interactions, multiple viroids in various citrus hosts show complicated antagonistic or synergistic relationships that lead to different symptoms, canopy volumes, fruit yields, and commercial performance; for instance, although co-infection by the two (CEVd and HSVd) viroids does not cause severe symptoms in citruses, their interaction is intriguing because of their high co-infection rate in the field and their identical biological properties in the same host [17,18].

Controlling exocortis and cachexia-xyloporosis diseases lacks chemical solutions. Prevention involves the use of viroid-free propagation material from certified sources, such as germplasm banks and greenhouses, rigorously tested through biological and molecular diagnostic assays [19]. Detection methods include nucleic acid technologies such as sequential polyacrylamide gel electrophoresis (S PAGE) and the widely used reverse transcription polymerase chain reaction (RT-PCR) [20]. Due to their adequate tolerance in high temperatures, the eradication of CEVd and HSVd from budwood through thermotherapy was successful by itself [9]. Despite the tolerance of viroids to heat, combining thermotherapy with a meristem tissue culture effectively produces virus/viroid-free plant material, as demonstrated in citruses and grapevines [21,22,23,24,25]. Thermotherapy combined with an in vitro meristem tip culture effectively eliminates viruses but requires the development of an efficient protocol for meristem growth, while the production of whole in vitro rooted plants is crucial for their successful ex vitro acclimatization in the greenhouse [26,27,28]. A plant tissue culture serves as a valuable biotechnological tool for the mass propagation of pathogen-free plants and establishment of germplasm banks, which are essential for controlling viral diseases, importing new cultivars, exchanging breeding material, preserving plant germplasm, and enhancing agricultural quality and yield while maintaining genetic stability and unique traits [26,27,28].

Considering all the above, the general aim and specific objectives of this study were to (1) establish an efficient in vitro propagation protocol for conserving and mass-producing this new important cultivar, (2) develop a simple and effective method to eliminate CEVd and HSVd viroids from *Citrus* x *limon* var. *limon* (L.) Burm. f. x *Citrus latifolia* var. *latifolia* using in vitro thermotherapy and a meristem tip culture along with reliable molecular detection assays (RT-PCR) for viroid-free plant production, and (3) produce an adequate number of viroid-free plants for establishing future mother plantations.

## 2. Materials and Methods

### 2.1. Disinfection of Plant Material and In Vitro Culture Conditions

Shoot tips and nodal segments with 2–3 buds of 1.5–2 cm in length were obtained from viroid-infected *Citrus* x *limon* var. *limon* (L.) Burm. f. x *Citrus latifolia* var. *latifolia* plants maintained at the greenhouse premises of the Institute of Plant Breeding and Genetic Resources of ELGO-DIMITRA, N. Greece. The disinfection protocol followed for shoot-tip and nodal-segment explants involved treatment with 0.07 g Signum fungicide in 100 mL ddH_2_O (15 min), followed by 70% alcohol (30 s) and 2% NaOCl (20 min) with five rinses using sterile ddH_2_O for each disinfectant (92% disinfection success). The successfully disinfected explants were cultured in vitro in the “Citrus P1” establishment medium for shoot proliferation consisting of the Murashige–Skoog (MS) nutrient medium [29] modified by its iron concentration (x 2FeEDTA) and supplemented with 0.5 mg L^−1^ benzyladenine (BA), 0.5 mg L^−1^ kinetin (KIN), 0.5 mg L^−1^ gibberellic acid (GA_3_), 0.25 mg L^−1^ α-naphthalene acetic acid (NAA), 30 g L^−1^ sucrose, and 6 g L^−1^ Plant Agar (pH 5.8). The incubation of explants was undertaken inside a growth chamber under specific environmental conditions (28 °C, 16 h photoperiod, White Fluorescent Light—WFL, 40 μmol m^−2^ s^−1^). Plant propagation material, which consisted of new proliferated shoot tips, was subcultured every 40 days (3 subcultures in a 120-day total culture period) in the “Citrus P1” medium within Magenta^TM^ vessels (Sigma-Aldrich, St. Louis, MO, USA, 200 mL) until a sufficient number of in vitro explants were produced for the establishment of the micropropagation protocol.

### 2.2. In Vitro Shoot Proliferation

Shoot tips, 1.5 cm in length, from previous in vitro proliferated cultures served as experimental material. Cytokinin effects, BA, or KIN at 0, 0.5, 1, and 2 mg L^−1^ in combination with 0.05 mg L^−1^ GA_3_ and 0.25 mg L^−1^ NAA were studied, with a positive control (only 0.5 mg L^−1^ GA_3_ + 0.25 mg L^−1^ NAA), negative control (without any plant growth regulator), and a low-concentration cytokinin mix (0.5 mg L^−1^ BA + 0.5 mg L^−1^ KIN + 0.5 mg L^−1^ GA_3_ + 0.25 mg L^−1^ NAA). The basal culture medium used was the modified MS (x 2FeEDTA) enriched with 30 g L^−1^ sucrose and 6 g L^−1^ Plant Agar, pH 5.8. Experimentation was conducted in Magenta^TM^ vessels with 25 mL of medium per vessel and lasted 30 days in the same growth chamber culture conditions as above. Parameters measured included shoot multiplication (%), shoot number per explant, shoot length (cm), proliferation rate, shoot fresh weight (FW), shoot dry weight (DW), shoot W/DW ratio, rooting percentage (%), root number per rooted microshoot, root length (cm), root FW (g), root DW (g), root FW/DW ratio, callus induction (%), and leaf chlorosis symptoms (%).

### 2.3. In Vitro Rooting

Shoot tips, 1.5 cm in length, from previous in vitro proliferated cultures served as experimental material. The effect of three (3) different auxin types, (1) indole-3-butyric acid (IBA), (2) α-naphthalene acetic acid (NAA), and (3) indole-3-acetic acid (IAA), each applied in four different concentrations (0, 0.5, 1 and 2 mg L^−1^), was studied. The basal culture medium used was the modified MS (x 2FeEDTA) enriched with 30 g L^−1^ sucrose and 6 g L^−1^ Plant Agar, pH 5.8. Experimentation was conducted in Magenta^TM^ vessels with 25 mL of medium per vessel and lasted 30 days in the same growth chamber culture conditions as above. After 30 days of culture, measurements were taken regarding the following parameters: rooting percentage (%), root number/rooted microshoot, root length (cm), root FW (g), root DW (g), root FW/DW ratio, shoot height (cm), shoot FW (g), shoot DW (g), and shoot FW/DW ratio.

### 2.4. Acclimatization and Production of Mother Plants

In late spring, in vitro rooted plants (*n* = 54 in total), derived from the different auxin type-concentration combination media, were transplanted into multi-position crates (200 mL per plant) with a peat (Terrahum)/perlite/vermiculite substrate mixture (2:1:1 *v*/*v*) and placed in a heated mist system in the greenhouse (18–21 °C base temperature, 15–25 °C air temperature, and 70–85% relative humidity). After 30 days of maintenance in the greenhouse mist, the survival rate (%) of the plants was recorded. Following that, the plants were moved to a greenhouse bench with sprinkler irrigation, experiencing increased light intensity and temperature but reduced humidity (mid-summer). After an additional 30 days, successfully acclimatized plants were transplanted into larger 0.33 L containers with a peat (KTS2)/perlite mixture (3:1 *v*/*v*) and placed on a greenhouse table bench under the same conditions. After an additional period of 60 days in mid-autumn and 120 days in early winter, successive transplants to larger pots (1 and 2.5 L) with a peat/perlite/soil mixture (2:1:½ *v*/*v*) were conducted for long-term ex situ conservation of the plants in an unheated greenhouse basin. The entire ex vitro acclimatization process took six months (180 days), from late spring to the middle of winter.

### 2.5. In Vitro Thermotherapy and Meristem Tip Culture for Viroid Elimination

#### 2.5.1. In Vitro Thermotherapy

Shoot tips and nodal segments with 2–3 buds of 1.5–2 cm in length from viroid-infected greenhouse plants were successfully established in vitro after disinfection (free from fungi and bacteria) and used as explants. The explants were cultured in the “Citrus P1” medium, placed in a growth chamber under a 16 h photoperiod and WFL of 40 μmol m^−2^ s^−1^, and subjected to thermotherapy process. During the 6-week total thermotherapy period, the temperature was gradually increased by 2 °C per week (28, 30, 32, 34, 36, and 38 °C). Macroscopic observations of stress and regeneration (i.e., new shoots induction, proliferation) capacity, along with weekly measurements of survival rate, and percentage of explants with stress symptoms (i.e., shoot-tip drying, leaf chlorosis, cut-off of shoot-base leaves, hyperhydricity, universal chlorosis, drying, and browning), were recorded (modified protocol) [21,22].

#### 2.5.2. In Vitro Meristem Tip Culture

Meristem tips of 0.5–1 mm were excised from new induced shoot tips from both explant types that survived thermotherapy without visible stress symptoms. A total of *n* = 46 meristems, each decapitated from a separate shoot tip, were cultured for 60 days in glass test tubes (25 mm width × 100 mm height), each containing 10 mL of the “Citrus P1” medium, inside a growth chamber of a 16 h photoperiod, WFL of 40 μmol m^−2^ s^−1^, and a temperature of 28 °C. At this stage, the first RT-PCR was employed in the 60-day differentiated meristems into new developed shoot tips to detect the presence of CEVd and HSVd viroids. The in vitro samples (*n* = 3 codes), cultured on the “Citrus P1” medium, that tested negative for both viroids (CEVd free, HSVd free) in the first RT-PCR were subsequently transferred to the optimized rooting medium [MS (x 2FeEDTA) + 30 g L^−1^ sucrose + 1 mg L^−1^ NAA + 6 g L^−1^ Plant Agar (pH 5.8)]. After a 30-day culture period, a second RT-PCR was conducted on the in vitro rooted plants to confirm the absence of the two viroids. In vitro rooted plants free from both viroids were then successfully acclimatized to ex vitro greenhouse conditions following the previously described procedure. Following a 6-month acclimatization period in the greenhouse (late spring–mid-winter), a third RT-PCR test was conducted on the greenhouse plant material to validate the sanitization (CEVd and HSVd free) of the propagating material.

#### 2.5.3. RT-PCR Detection of CEVd and HSVd

Various RT-PCR techniques can be applied for the detection and subsequent characterization of the viroids owing to their small size, such as the multiplex one-step RT-PCR, which is an effective tool for rationalizing the concurrent detection of up to five citrus viroids, including CEVd and HSVd, minimizing time and labor barren of influencing sensitivity and specificity [30]. In the present study, total RNA was extracted from plant material (leaves with stem) following the protocol of Chatzinasiou et al. [31] modified by Maliogka et al. [32]. RNA quality was assessed using one-tube RT-PCR, amplifying a 194 bp sequence of the ubiquitin gene [33]. For HSVd detection, primers targeting a 173 nt region were used in one-tube RT-PCR as described by Maliogka et al. [32]. CEVd detection took place employing primers targeting a 373 nt region and a protocol from Wang et al. [34] to amplify the whole viroid genome. Amplicons were analyzed by electrophoresis on a 1.5% agarose gel stained with Midori Green Advance gel (Nippon, Dueren, Germany) and visualized under UV light.

### 2.6. Statistical Analysis

Statistical analysis for the in vitro shoot proliferation and rooting experiments involved completely randomized designs. IBM^®^ SPSS^®^ Statistics Version 21.0 was used with ANOVA and a significance level of 5%, applying Tukey’s multiple range test criterion. The shoot proliferation experiment comprised 9 treatments with 20 replicates each, while the rooting experiment had 12 treatments with 20 replicates each (4 vessels/treatment × 5 explants/vessel). One-way ANOVA was employed for means comparison in the shoot proliferation experiment. The rooting experiment, designed as a 3 × 4 multivariate, considered three auxin types (IBA, NAA, IAA) and four concentrations (0, 0.5, 1, 2 mg L^−1^) per auxin type; in addition, the main effects and their interaction were analyzed using General Linear Model and two-way ANOVA. Each experiment was conducted in triplicate.

## 3. Results

### 3.1. In Vitro Shoot Proliferation

**Figure 1:**
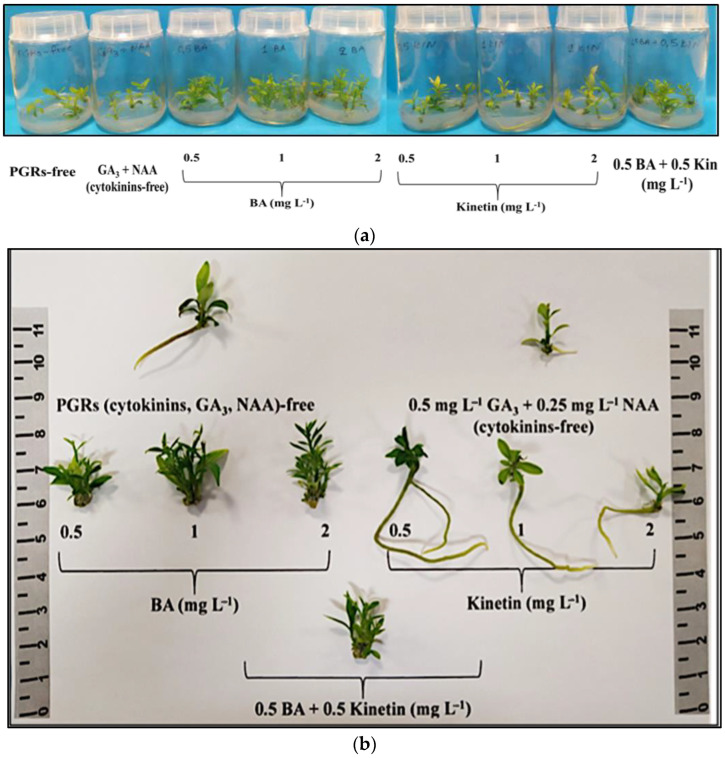
In vitro shoot proliferation and/or rooting of *Citrus* x *limon* var. *limon* (L.) Burm. f. x *Citrus latifolia* var. *latifolia*) after 30 days of culture in modified MS (x 2FeEDTA) medium enriched with 30 g L^−1^ sucrose and 6 g L^−1^ Plant Agar under different types (ΒA, ΚΙΝ) and concentrations (0, 0.5, 1, 2 mg L^−1^) of cytokinins applied individually and combined with 0.5 mg L^−1^ GA_3_ + 0.25 mg L^−1^ NAA: (**a**) inside culture vessels; (**b**) outside culture vessels.

The negative control [plant growth regulators (PGRs)-free] did not induce multiple shoots, unlike the other eight treatments showing proliferation. The most effective treatments for shoot multiplication (80–90%) were 0.5 mg L^−1^ BA, 1 mg L^−1^ BA, 2 mg L^−1^ BA, and 0.5 mg L^−1^ BA + 0.5 mg L^−1^ KIN. The 0.5–2 mg L^−1^ BA treatments resulted in a higher shoot number (3.00–3.45 shoots/explant) and proliferation rate (3.35–4.05). Shoot length was significantly greater (1.66–1.76 cm) with 0.5 mg L^−1^ BA or 0.5 mg L^−1^ BA + 0.5 mg L^−1^ KIN. Shoot FW and DW were notably higher with the combined application of the two cytokinins (0.5 mg L^−1^ BA + 0.5 mg L^−1^ KIN). Thus, 0.5–2 mg L^−1^ BA + 0.5 mg L^−1^ GA_3_ + 0.25 mg L^−1^ NAA was the most effective treatment for initial multiple shoot induction, while 0.5 mg L^−1^ BA + 0.5 mg L^−1^ KIN + 0.5 mg L^−1^ GA_3_ + 0.25 mg L^−1^ NAA was optimal for the subsequent elongation of the produced microshoots (Table S1, Figure 1a,b).

Root formation occurred at a rate of 5–15% in the positive control (cytokinin free) and with the addition of KIN to the medium irrespective of concentration (0.5–2 mg L^−1^). Notably, KIN at 0.5 mg L^−1^ yielded superior rooting results with two roots per rooted microshoot, an average length of 6.95 cm, higher root fresh and dry biomass, and a lower FW/DW ratio. Callus induction was observed in explants exposed to BA or KIN or 0.5 mg L^−1^ BA + 0.5 mg L^−1^ KIN in a rate of 10–90%, contrasting with the control treatments (positive, negative), where no callusing occurred. BA (in any concentration) and 0.5 mg L^−1^ BA + 0.5 mg L^−1^ KIN showed higher callus induction rates (70–90%) compared to KIN alone (10–60%). Mild leaf chlorosis stress symptoms were observed in 25–50% of the explants cultured in medium enriched with KIN in the two higher concentrations of 1 mg L^−1^ (25%) and 2 mg L^−1^ (50%) (Table S2, Figure 1a,b).

### 3.2. In Vitro Rooting and Acclimatization

**Figure 2:**
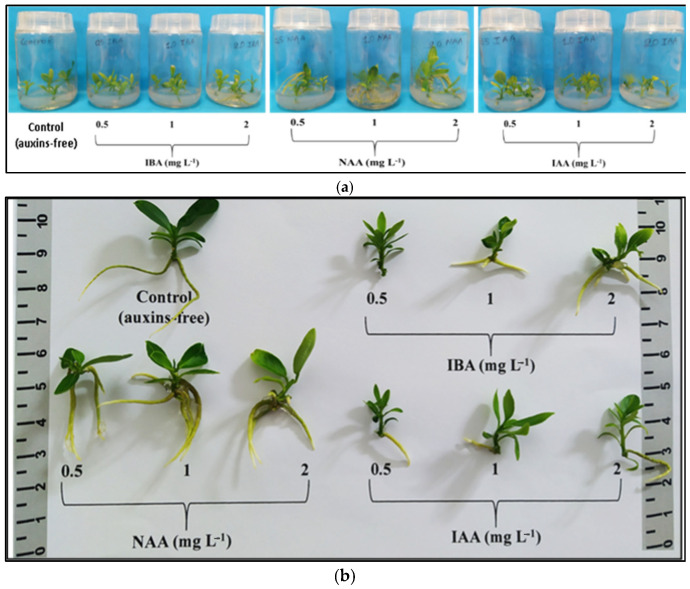
The in vitro rooting and vegetative growth of *Citrus* x *limon* var. *limon* (L.) Burm. f. x *Citrus latifolia* var. *latifolia* after 30 days of culture in a modified MS (x2FeEDTA) nutrient medium enriched with 30 g L^−1^ sucrose and 6 g L^−1^ Plant Agar under the effect of different types (IBA, NAA, IAA) and concentrations (0, 0.5, 1, 2 mg L^−1^) of auxins: (**a**) inside culture vessels; (**b**) outside culture vessels.

The number of roots was significantly increased (3.20–3.75 roots/rooted microshoot) with NAA (0.5–2 mg L^−1^) and the highest IBA concentration of 2 mg L^−1^. Root length was significantly improved (3.90 cm) with 2 mg L^−1^ IAA. Substantial increases in root FW and DW were observed when the medium included 1 mg L^−1^ NAA. The highest rooting percentage (95%) occurred with 2 mg L^−1^ NAA, followed by a significantly lower percentage (80%) with 1 mg L^−1^ NAA. Considering all individual rooting parameters, NAA at 1 mg L^−1^ proved to be the most suitable auxin for the in vitro rooting stage. The 2 mg L^−1^ NAA treatment, despite having the highest rooting percentage (95%), was not selected due to the presence of mild stress symptoms in the explants, including universal leaf chlorosis, the browning of the microshoot base, mild browning, and the leaf abscission of the upper shoot segment (Table S3, Figure 2a,b).

Shoot height was significantly increased (2.03 cm) with 0.5 mg L^−1^ IBA. Both IBA and IAA, regardless of concentration, significantly increased shoot height compared to the control, unlike NAA, which showed similar results. NAA at 1 mg L^−1^ yielded the highest shoot biomass values (FW, DW), and the shoot FW/DW ratio was significantly increased (undesirable trait) under 2 mg L^−1^ NAA (Table S4, Figure 2a,b).

**Figure 3:**
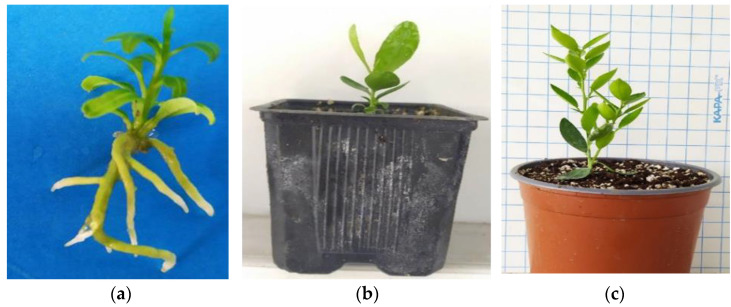
The ex vitro acclimatization of in vitro rooted plants to the greenhouse and further vegetative growth of plants in a 6-month period from late spring to mid-winter (consecutive transplants into larger pots, substrate mixture growth-dependent) in *C.* x *limon* var. *limon* (L.) Burm. f. x *C. latifolia* var. *latifolia*: (**a**) the in vitro rooted plant; (**b**) the ex vitro acclimatized plant in a 0.33 L pot; (**c**) the growth of the acclimatized plant in a 2.5 L pot.

After a 6-month (180 days) maintenance period in the greenhouse extending from late spring to the middle of winter (30 days in mist + 30 days in automated irrigation bench + 120 days in basin manually irrigated), the in vitro rooted plantlets achieved an 85% survival rate, with 46 out of 54 plants successfully acclimatizing ex vitro (Figure 3a–c).

### 3.3. In Vitro Thermotherapy and Apical Meristem Culture

**Figure 4:**
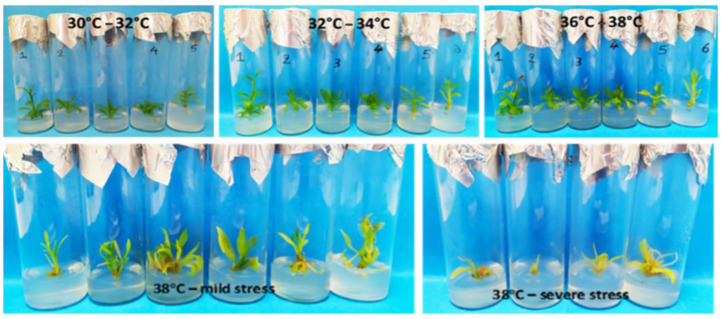
Vegetative growth and development and appearance of stress symptoms in shoot-tip explants during in vitro thermotherapy at successively increasing temperature regimes in *Citrus* x *limon* var. *limon* (L.) Burm. f. x *Citrus latifolia* var. *latifolia*.

For the first explant type (shoot tips) following thermotherapy at 38 °C, a 90% survival rate was achieved, with 32% exhibiting no visible stress symptoms. Among the explants with stress symptoms, complete recovery was observed for 30% when transferred to a growth chamber at 28 °C. The gradual temperature increase up to 36 °C did not induce stress symptoms, but proliferation ceased at 36 °C. Maximum proliferation and vegetative growth occurred at 34 °C, prompting a subculture at 36 °C with reduced BA concentration (0.2 mg L^−1^) to address hyperhydricity due to elevated temperatures (Figure 4, Table 1).

For the second explant type (shoot nodal segments) following thermotherapy at 38 °C, there was a 76% average survival rate (19/25). Among the surviving explants, 24% (6/25) exhibited new shoot induction, while 40% (10/25) displayed stress symptoms. Explants with stress symptoms were less resistant, showing less vegetative development during each temperature alteration range of in vitro thermotherapy. In contrast, the more developed explants not only survived but also demonstrated significant regrowth, producing multiple new shoots on the initial shoot (Table 1).

**Figure 5:**
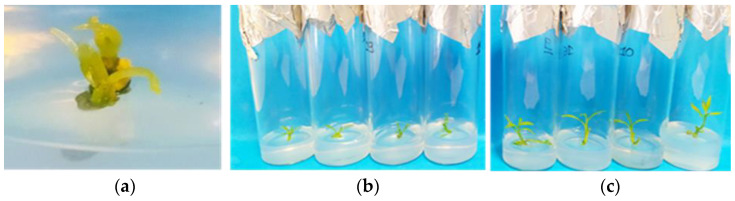
In vitro differentiation and development of apical meristems (0.5–1 mm) into new complete shoot tips in modified MS (x 2Fe) medium enriched with 30 g L^−1^ sucrose, 0.5 mg L^−1^ BA, 0.5 mg L^−1^ KIN, 0.5 mg L^−1^ GA_3_, 0.25 mg L^−1^ NAA (pH 5.8), and 6 g L^−1^ Plant Agar in *Citrus* x *limon* var. *limon* (L.) Burm. f. x *Citrus latifolia* var. *latifolia*: (**a**) growth of apical meristem after one week of culture; (**b**) after 30 days; (**c**) after 60 days.

Out of the shoot-tip and shoot nodal-segment explants that survived thermotherapy, 90% and 76%, respectively, apical meristems of 0.5–1 mm were cut from the new induced lateral shoot tips regardless of the initial explant type. The meristems exhibited a 100% survival rate and successfully regenerated into new shoot tips after a 60-day culture (Figure 5a–c).

**Figure 6:**
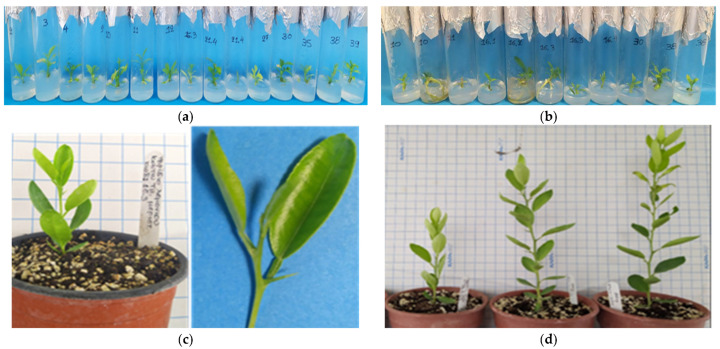
Plant material samples tested by RT-PCR for CEVd and HSVd in *Citrus* x *limon* var. *limon* (L.) Burm. f. x *Citrus latifolia* var. *latifolia*: (**a**) 14 in vitro shoot tips cultured in MS (x 2Fe) + 30 g L^−1^ sucrose + 0.5 mg L^−1^ BA + 0.5 mg L^−1^ KIN + 0.5 mg L^−1^ GA_3_ + 0.25 mg L^−1^ NAA (pH 5.8) + 6 g L^−1^ Plant Agar (1st round RT-PCR); (**b**) 6 in vitro rooted shoot tips cultured in MS (x 2Fe) + 30 g L^−1^ sucrose + 1 mg L^−1^ NAA (pH 5.8) + 6 g L^−1^ Plant Agar (2nd round RT-PCR); (**c**) 6-month ex vitro acclimatized greenhouse plant sample (code: Lime 16.3) (3rd round RT-PCR); (**d**) subsequent vegetative growth of acclimatized viroid-free plant under greenhouse conditions during late spring–mid-winter.

In the 1st RT-PCR round on the 60-day in vitro cultured and differentiated meristems into new shoot tips, it was found that 43% (6/14) were free only of CEVd, 21% (3/14) were free only of HSVd, and 21% (3/14) tested negative for both viroids (sample codes: Lime 11, Lime 16.3, Lime 30) (Figure 6a, Table 2). The 2nd RT-PCR viroid detection results revealed that 33% (2/6) (sample codes: Lime 16.3, Lime 30) of the in vitro rooted shoot-tip samples were negative for both CEVd and HSVd, 50% (3/6) were negative only of CEVd, and 33% (2/6) were free only of HSVd (Figure 6b, Table 2). After 6 months of ex vitro acclimatization, a 3rd RT-PCR confirmed viroid-free status (CEVd and HSVd) in ex vitro greenhouse plants, originated from in vitro material (sample code: Lime 16.3), demonstrating success in sanitization (Figure 6c,d, Table 2).

**Figure 7:**
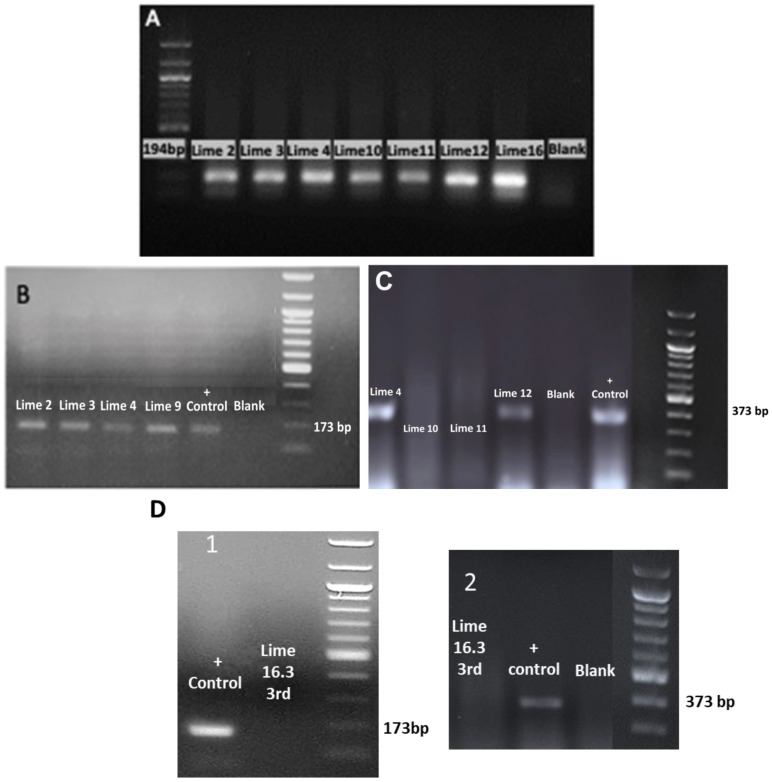
The agarose gel electrophoretic analysis of RT-PCR products obtained using (**A**) the primers (22) targeting the ubiquitin gene (internal control) [33]; (**B**) the primers targeting the HSVd [32]; (**C**) The primers targeting CEVd [34]; (**D**) the agarose gel electrophoretic analysis of the 3rd round of RT-PCR in the ex vitro acclimatized greenhouse sample plant (code: Lime 16.3) after thermotherapy and tissue culture rescue for HSVd (1) and CEVd (2).

RT-PCR assays successfully detected CEVd and HSVd (Figure 7A–D). The agarose gel electrophoretic analysis of RT-PCR products obtained using the primers 22 targeting the ubiquitin gene (internal control) (Figure 7A), the primers targeting HSVd (Figure 7B), the primers targeting CEVd (Figure 7C), and 3rd RT-PCR showed CEVd and HSVd elimination in the ex vitro acclimatized greenhouse plants (sample code: Lime 16.3) (Figure 7D).

## 4. Discussion

In this study, a novel in vitro viroid elimination protocol for *C.* x *limon* var. *limon* (L.) Burm. f. x *C. latifolia* var. *latifolia* was developed. Unlike traditional methods involving growth chambers, the described approach utilizes in vitro thermotherapy and an apical meristem culture for a faster and more cost-effective elimination of CEVd and HSVd. This method ensures efficient multiplication in vitro, followed by the thermotherapy of shoot tips and shoots from nodal segments. The process allows for in vitro rooting, successful acclimatization in the greenhouse, and a quicker detection of citrus viroids. This alternative method offers advantages such as a cheaper diagnosis and reduced time for the production of viroid-free citrus propagating material. Beyond addressing sanitary concerns, this study emphasizes the importance of the conservation of *Citrus* species with superior quality traits, utilizing biotechnological tools such as the in vitro culture for sustainable agriculture and plant breeding to rapidly introduce improved plants for medicinal purposes [20,35,36].

Meristems can be free of viruses/viroids due to (1) rapid cell division inhibiting viral replication [37]; (2) RNA silencing in plant defense preventing virus accumulation [38]; (3) the slow cell-to-cell spread of viruses, mainly through the vascular system absent in meristems [39,40]; and (4) a potential link between virus presence and plasmodesmata development, observed with fewer occurrences in virus-free tissues [33]. Selecting the right temperature and exposure time is crucial for balancing virus degradation and plant damage [41]. Higher temperatures and longer exposure durations, typically 35–42 °C for 4–6 weeks, increase virus-eradication frequency depending on virus type, plant species, and the virus–host combination [42]. Although a temperature range of 32–40 °C has been used, 37–38 °C is most commonly applied in thermotherapy experiments [43]. Success also hinges on factors such as plant genotype, virus/viroid species, their localization, interaction with plants, treatment conditions, and meristem size (smaller meristems have lower survival rates but higher virus elimination efficiency) [25].

Thermotherapy aims to maximize plant survival and regeneration while maintaining antiviral efficacy. In general, regeneration rates are lower than survival rates [44]. In this study, 38 °C thermotherapy resulted in 90% and 76% survival rates for shoot tips and nodal-segment shoots, with 100% regeneration from surviving meristems after 60 days. Since viroids can withstand high temperatures and increase their concentration, the main factor for their elimination is an in vitro meristem tip culture, followed by the impact of in vitro thermotherapy (the former primarily targets viruses) [45]. The lower elimination rates for HSVd compared to CEVd in this study may be due to the higher accumulation and putative faster movement of HSVd in the limonime hybrid compared to CEVd [46]. In *Citrus* spp., in vitro tissue cultures completely eliminated CEVd and showed eradication rates of 60–100% for HSVd [11,47,48]. Complex interactions, including antagonism and synergy, may occur between CEVd and HSVd when they are found in mixed infections in the same citrus host. Despite similar biological functions and shared cellular and subcellular spaces, their relationship is intricate [46]. In specific tissues of two citrus cultivars (blood orange and Murcott mandarin), a significant increase in the CEVd and HSVd population has been observed under mixed-infection conditions compared to their concentrations under single-infection conditions, showing a positive correlation between CEVd and HSVd in terms of titer enhancement, localization similarity, and a lack of symptom aggravation under mixed-infection conditions [17,18].

Various thermotherapy methods aim to achieve a high proportion of surviving plants with strong regeneration ability while maintaining antiviral efficacy; however, plants undergoing thermotherapy experience significant stress [49], leading to lower regeneration rates compared to survival rates [44]. In this study, 90% and 76% survival rates were observed after thermotherapy, with 68% and 40% exhibiting stress symptoms, respectively. From the surviving explants, 100% regeneration and survival rates of meristems into new shoot tips were achieved after 60 days of culture. The temperature-dependent processes of virus/viroid multiplication, inactivation, and movement in plants, coupled with the influence of virus/viroid type and host genotype, can impact the effectiveness of thermotherapy [50].

Specific molecular assays, namely RT-PCRs, which exhibit high detection sensitivity, were applied herein in order to achieve reliable viroid detection. The application of RT-PCR assays in three different time points unveiled the absence of CEVd and HSVd in a number of in vitro tested plantlets as well as in the ex vitro acclimatized plants that were established in the screenhouse. Given that the effectiveness of thermotherapy alone in eliminating viroids is rather poor, as documented in other woody plants [45], it is assumed that the CEVd and HSVd elimination was mainly due to the cultivation of the apical meristem from the infected plants [51]. It has been shown that potato spindle tuber viroid (PSTVd) exclusion from the meristem is possibly due to RNA silencing [52]. Moreover, it is established that the RNA silencing-mediated plant defense against viruses and viroids is temperature dependent [53]. Whether thermotherapy, through its possible effect on RNA silencing mechanism, could further exclude CEVd and HSVd from the meristematic tissues of the tested citrus genotype remains to be unveiled. Further testing of the ex vitro grown plant material in a later stage of development is needed before it can be used for the establishment of mother plantations.

In this study, 0.5–2 mg L^−1^ BA was most effective for initial multiple shoot induction, while 0.5 mg L^−1^ BA + 0.5 mg L^−1^ KIN performed best during shoot elongation. This response is linked to the linear relationship that exists among various factors, including concentrations and types (cytokinins, auxins) of PGRs, genotype, age, and explant type [54,55]. The cytokinin/auxin ratio is crucial for shoot proliferation, with BA and kinetin being potent cytokinins for *Citrus* spp., with species-dependent concentrations [56]. GA_3_, besides influencing plant height, occasionally enhances in vitro shoot induction and multiplication, particularly when combined with BA [26,57]. Discrepancies in shoot induction and proliferation may be PGR type and concentration dependent, influenced by variations in identification or action mechanisms, cytokinin characteristics, and the competitive interplay between cytokinin and auxin in the medium [58,59]. This study found BA superior to KIN for shoot proliferation, consistent with previous *Citrus* species studies using various PGR combinations [4,60,61].

Various auxins (i.e., IBA, NAA, and IAA) can be used for in vitro rooting, with NAA proving to be the most effective in promoting rooting in *Citrus* species and rootstocks. NAA accelerates the root initiation process, increases rooting rates, and enhances plantlet survival under ex vitro conditions [4,62]. The superior effectiveness of NAA on root number, shoot and root biomass yields, and shoot height may be attributed to its higher stability, faster transport, and shorter persistence period within plant tissues compared to IBA and IAA [63,64]. In this study, 1 mg L^−1^ NAA was selected as the most beneficial treatment, resulting in 80% rooting. Despite the need for higher auxin concentrations in some plant species, caution is required to avoid toxicity due to increased ethylene synthesis [59].

The success of a micropropagation protocol for in vitro plant propagation relies on the efficient transfer of plants to ex vitro conditions with a high survival rate [61]. In the studied lemon x lime hybrid, the ex vitro survival rate of rooted microshoots in the greenhouse mist was 85% regardless of the in vitro rooting culture medium. The 15% survival loss observed may be attributed to water stress and physiological changes during the acclimatization stage. Other *Citrus* species have shown high ex vitro survival rates (60–100%) with different substrate mixtures [61,63,65,66,67,68].

In vitro somatic embryogenesis, from both style and stigma cultures, has been demonstrated to be a loftily efficient sanitation process resulting in the thorough expungement of the main virus/viroid and virus/viroid-like diseases related to citruses for the production of healthy plants [47,69,70]. This technique was implemented to eradicate CEVd and HSVd from some *Citrus* species [47]. For instance, somatic embryogenesis in 13 genotypes of two different *Citrus* species, lemons and sweet oranges, infected by either CEVd or HSVd led to the elimination of CEVd in 12 out of the 13 tested genotypes; nevertheless, there was evidence that HSVd was the most infectious viroid because it was eliminated only from 5/13 tested genotypes [47]. Sanitation by in vitro shoot-tip grafting has also been established to be a highly potent method for the citrus graft-transmissible disease eradication of citrus viroids (success rate of about 100%). For example, it has been stated that CEVd and HSVd can be regularly eliminated from citruses by shoot-tip grafting [71,72]. Due to the extraordinary tolerance of citrus viroids to heat, the application of thermotherapy as a sanitary method was not sufficient to eliminate viroids from citrus budwoods at a successful rate. Notwithstanding, CEVd and HSVd, similar to all citrus viroids, appear to be eliminated from propagative material by shoot-tip grafting or by the evolvement of nucellar budlines [9]. Nonetheless and despite the incapacity of the majority of viruses to attack the youngest cells in the shoot apex, some viroids can infect all plant cells and do not withdraw from viroid-free areas or tissues in diseased plants [73]. It has been reported that an alternative solution for the removal of viroids is the application of the cold therapy method, which prevents their movement and replication in the apical meristem, resulting in viroid-free cells and tissues [73,74,75]. A leaf primordia-free meristem culture and shoot-tip cryotherapy, predominantly the combinational scheme of long-term cold therapy and a meristem culture [76,77,78,79], are a recent effective virus and viroid eradication technology. The results of this study contribute an additional method for developing a viroid-free citrus hybrid and for conservation. Because the concentration of viroids, unlike viruses, increases at high temperatures, techniques such as a meristematic culture, cryotherapy, or somatic embryogenesis applied individually and in combinations may be more effective than thermotherapy, an issue that merits investigation as a future perspective. In an upcoming work, the consideration of whether meristem tips of control shoots that have not undergone heat stress will also be investigated and tested for viroid presence for obtaining viroid-free material in the studied citrus hybrid. This would either render the viroid elimination protocol potentially more facile or conversely provide a rationale for the prerequisite of the heat treatment for a higher success rate.

## 5. Conclusions

The work presented in this manuscript has its novelty, especially on the micropropagation of a new limonime hybrid and the in vitro thermotherapy assessment combined with the in vitro meristem tip culture, for the elimination of viroids in a citrus species, differing from other citrus micropropagation studies in other citrus species, including lemons (*Citrus limon*) or Persian/Tahiti limes (*Citrus latifolia*), among others. In vitro thermotherapy (28–38 °C, 5 weeks, 2 °C increase/week) resulted in high explant survival rates (76–100%). The regeneration of meristems into new shoot tips achieved 100% success after 60 days of culture in modified MS (x2Fe) + 0.5 mg L^−1^ BA + 0.5 mg L^−1^ KIN + 0.5 mg L^−1^ GA_3_ + 0.25 mg L^−1^ NAA. The 21–33% of in vitro cultured explants tested negative for both CEVd and HSVd viroids. HSVd was more challenging to eradicate than CEVd. The combined method of in vitro thermotherapy and the meristem tip culture demonstrated a sufficient success rate in eliminating CEVd and HSVd viroids in the in vitro plants and the complete sanitation of the ex vitro greenhouse plant propagating material. A successful micropropagation protocol was developed for *C.* x *limon* var. *limon* x *C. latifolia* var. *latifolia*. The optimal medium for in vitro shoot proliferation was the modified MS (x2Fe) with 0.5–2 mg L^−1^ BA + 0.5 mg L^−1^ GA_3_ + 0.25 mg L^−1^ NAA, while for in vitro rooting, it was the modified MS (x2Fe) + 1 mg L^−1^ NAA. The ex vitro survival rate of the in vitro rooted explants in the greenhouse was high (85%). Further studies using functional genomics, such as a transcriptomic or proteomic analysis, can provide a deeper understanding of the complex mechanisms involved in citrus–CEVd/HSVd interaction. This knowledge could lead to the development of innovative management strategies for these viroids, enhancing plant protection in citruses.

## Figures and Tables

**Table 1:** Effect of in vitro thermotherapy on percentages (%) of survival and stressed explants in *Citrus* x *limon* var. *limon* (L.) Burm. f. x *Citrus latifolia* var. *latifolia*.

Explant Type	T (°C)	Nutrient Culture Medium	Survival of Explants (%)	Stressed Explants (%)	Stress Symptoms in Explants during In Vitro Thermotherapy
Shoot tips	28	Citrus P1 (subculture)	100% (40/40)	0% (0/40)	38 °C: - 30% (12/40) (mild) [shoot-tip drying, leaf chlorosis, cut off of shoot-base leaves] - 28% (11/40) (severe) [hyperhydricity, shoot-tip drying, universal chlorosis, mild browning of explants] - 10% (4/40) [complete necrosis] - 32% (13/40) no symptoms
	30	Citrus P1	100% (40/40)	0% (0/40)	
	32	Citrus P1	100% (40/40)	0% (0/40)	
	34	Citrus P1	100% (40/40)	0% (0/40)	
	36	Citrus P2 (subculture)	100% (40/40)	0% (0/40)	
	38	Citrus P2	90% (36/40)	68% (27/40)	
Shoot nodal segments	28	Citrus P1	100% (25/25)	0% (0/25)	32 °C: Mild hyperhydricity 34 °C: Severe hyperhydricity, necrosis 38 °C: - 32% (8/25), shoot base/tip drying, no proliferation - 8% (2/25), complete drying, browning, necrosis - 44% (11/25), green-colored non-stressed explants of which 24% with proliferation
	30	Citrus P1	100% (25/25)	0% (0/25)	
	32	Citrus P1	100% (25/25)	32% (8/25)	
	34	Citrus P1	84% (21/25)	32% (8/25)	
	36	Citrus P1	84% (21/25)	32% (8/25)	
	38	Citrus P1	76% (19/25)	40% (10/25)	

**Table 2:** Detection results of HSVd and CEVd in in vitro plant samples (1st + 2nd RT-PCR) and ex vitro acclimatized greenhouse samples (3rd RT-PCR) in *C.* x *limon* var. *limon* (L.) Burm. f. x *C. latifolia* var. *latifolia*.

1st RT-PCR (In Vitro)			2nd RT-PCR (In Vitro)			3rd RT-PCR (Greenhouse)		
Sample Code	HSVd	CEVd	Sample Code	HSVd	CEVd	Sample Code	HSVd	CEVd
Lime 2	+	+						
Lime 3	+	−						
Lime 4	+	+						
Lime 9	+	+						
Lime 10	+	−	Lime 10	+	−			
Lime 11	−	−						
Lime 12	+	+	Lime 12	+	+			
Lime 16.3	−	−	Lime 16.3	−	−	Lime 16.3	−	−
Lime 21	+	+						
Lime 27	+	+	Lime 27	+	+			
Lime 30	−	−	Lime 30	−	−			
Lime 35	+	+						
Lime 38	+	−	Lime 38	+	+			
Lime 39	+	+						

RT-PCR (1st round, *n* = 14, 60-day culture, optimized in vitro proliferation medium/2nd round, *n* = 6, 30-day culture, optimized in vitro rooting medium/3rd round, *n* = 1, 6 months, ex vitro, greenhouse).

## Data Availability

The original contributions presented in the study are included in the article/Supplementary Material; further inquiries can be directed to the corresponding author.

## Supplementary Materials

The following supporting information can be downloaded at https://www.mdpi.com/article/10.3390/biotech13030037/s1, Table S1: Effect of different cytokinin types (ΒA, ΚIN) and concentrations (0, 0.5, 1, 2 mg L^−1^) applied in various combinations with 0.5 mg L^−1^ GA_3_ + 0.25 mg L^−1^ NAA on in vitro shoot proliferation parameters of *Citrus* x *limon* var. *limon* (L.) Burm. f. x *C. latifolia* var. *latifolia*, Table S2: Effect of different cytokinin types (ΒA, ΚIN) and concentrations (0, 0.5, 1, and 2 mg L^−1^), applied individually and combined with 0.5 mg L^−1^ GA_3_ + 0.25 mg L^−1^ NAA, on in vitro rooting parameters of *Citrus* x *limon* var. *limon* (L.) Burm. f. x *C. latifolia* var. *latifolia*, Table S3: Effect of different auxin types (IBA, NAA, IAA) and concentrations (0, 0.5, 1, 2 mg L^−1^) on in vitro rooting parameters of *Citrus* x *limon* var. *limon* (L.) Burm. f. x *C. latifolia* var. *latifolia*, Table S4: Effect of different auxin types (IBA, NAA, IAA) and concentrations (0, 0.5, 1, 2 mg L^−1^) on in vitro vegetative growth parameters of *Citrus* x *limon* var. *limon* (L.) Burm. f. x *C. latifolia* var. *latifolia*.
