# Supplementary material for: The Elimination of Viroids through In Vitro Thermotherapy and a Meristem Tip Culture from a New Limonime Hybrid (Citrus x limon var. limon (L.) Burm. f. x Citrus latifolia var. latifolia)
Source: BioTech (Basel). 2024 Sep 23;13(3):37. doi: 10.3390/biotech13030037 (PMC11430235; doi:10.3390/biotech13030037)
Supplement: Supplementary file 1 [file biotech-13-00037-s001.zip › biotech-3191303-supplementary.pdf]

BioTech MDPI

Supplementary Materials

The Elimination of Viroids through In Vitro Thermotherapy and a Meristem Tip Culture from a New Limonime Hybrid (*Citrus* x *limon* var. *limon* (L.) Burm. f. x *Citrus latifolia* var. *latifolia*)

Virginia Sarropoulou, Katerina Grigoriadou, Varvara I. Maliogka, Chrysoula-Lito Sassalou and Vasileios Ziogas

Table S1. Effect of different cytokinin types (BA, KIN) and concentrations (0, 0.5, 1, 2 mg L^{-1}) applied in various combinations with 0.5 mg L^{-1} GA_3 + 0.25 mg L^{-1} NAA on *in vitro* shoot proliferation parameters of *Citrus* x *limon* var. *limon* (L.) Burm. f. x *C. latifolia* var. *latifolia*

Treatments (mg L^{-1})				Shoot multiplication (%)	Shoot number/ explant	Shoot length (cm)	Proliferation rate	Shoot FW (g)	Shoot DW (g)	Shoot FW/DW ratio
BA	KIN	GA_3	NAA							
0	0	0	0	0 ± 0 d	1.00 ± 0.00 c	1.35 ± 0.08 b	1.10 ± 0.07 c	0.067 ± 0.005 d	0.022 ± 0.002 d	3.07 ± 0.01 i
0	0	0.5	0.25	20 ± 14 bc	1.40 ± 0.20 c	1.38 ± 0.08 b	1.50 ± 0.26 c	0.067 ± 0.007 d	0.021 ± 0.002 d	3.27 ± 0.02 h
0.5	0	0.5	0.25	90 ± 6 a	3.15 ± 0.35 ab	1.66 ± 0.06 a	3.55 ± 0.35 ab	0.214 ± 0.023 a	0.047 ± 0.005 ab	4.57 ± 0.01 c
1	0	0.5	0.25	80 ± 8 a	3.00 ± 0.36 ab	1.56 ± 0.09 ab	3.35 ± 0.39 ab	0.163 ± 0.021 b	0.033 ± 0.004 c	4.95 ± 0.02 b
2	0	0.5	0.25	90 ± 6 a	3.45 ± 0.29 a	1.55 ± 0.09 ab	4.05 ± 0.34 a	0.213 ± 0.016 a	0.040 ± 0.003 bc	5.27 ± 0.01 a
0	0.5	0.5	0.25	15 ± 5 bc	1.15 ± 0.08 c	1.58 ± 0.07 ab	1.35 ± 0.11 c	0.105 ± 0.009 cd	0.029 ± 0.003 cd	3.56 ± 0.01 e
0	1	0.5	0.25	30 ± 10 b	1.30 ± 0.11 c	1.50 ± 0.08 ab	1.45 ± 0.14 c	0.111 ± 0.010 cd	0.033 ± 0.003 c	3.38 ± 0.01 g
0	2	0.5	0.25	25 ± 10 bc	1.30 ± 0.13 c	1.57 ± 0.07 ab	1.55 ± 0.14 c	0.137 ± 0.016 bc	0.040 ± 0.005 bc	3.44 ± 0.01 f
0.5	0.5	0.5	0.25	80 ± 12 a	2.50 ± 0.25 b	1.76 ± 0.12 a	3.25 ± 0.32 b	0.234 ± 0.023 a	0.052 ± 0.005 a	4.52 ± 0.01 d
p-values (one-way ANOVA)				0.000***	0.000***	0.025*	0.000***	0.000***	0.000***	0.000***

Means ± standard errors (S.E.) with different letters in each column are statistically significantly different from each other based on Tukey's test at a 5% level ($p \leq 0.05$). * $p \leq 0.05$, *** $p \leq 0.000$ [30 days culture, modified MS (x 2FeEDTA) medium + 30 g L^{-1} sucrose + 6 g L^{-1} Plant Agar, pH 5.8].

Table S2. Effect of different cytokinin types (BA, KIN) and concentrations (0, 0.5, 1, and 2 mg L^{-1}), applied individually and combined with 0.5 mg L^{-1} GA_3 + 0.25 mg L^{-1} NAA on *in vitro* rooting parameters of *Citrus* x *limon* var. *limon* (L.) Burm. f. x *C. latifolia* var. *latifolia*

Treatments (mg L^{-1})				Rooting (%)	Root number / rooted microshoot	Root length (cm)	Root FW (g)	Root DW (g)	Root FW/DW	Callus induction (%)	Mild leaf chlorosis (%)
BA	KIN	GA_3	NAA								
0	0	0	0	10 ± 6 ab	1.00 ± 0.00 c	2.00 ± 0.10 d	0.028 ± 0.001 c	0.005 ± 0.000 d	6.08 ± 0.01 a	0 ± 0 d	0 ± 0 b
0	0	0.5	0.25	5 ± 5 ab	1.00 ± 0.00 c	1.50 ± 0.00 e	0.012 ± 0.000 d	0.002 ± 0.000 e	5.22 ± 0.00 b	0 ± 0 d	0 ± 0 b
0.5	0	0.5	0.25	0 ± 0 b	0.00 ± 0.00 d	0.00 ± 0.00 f	0.000 ± 0.000 e	0.000 ± 0.000 f	-	90 ± 6 a	0 ± 0 b
1	0	0.5	0.25	0 ± 0 b	0.00 ± 0.00 d	0.00 ± 0.00 f	0.000 ± 0.000 e	0.000 ± 0.000 f	-	70 ± 13 ab	0 ± 0 b
2	0	0.5	0.25	0 ± 0 b	0.00 ± 0.00 d	0.00 ± 0.00 f	0.000 ± 0.000 e	0.000 ± 0.000 f	-	80 ± 8. ab	0 ± 0 b
0	0.5	0.5	0.25	5 ± 5 ab	2.00 ± 0.00 a	6.95 ± 0.00 a	0.084 ± 0.000 a	0.030 ± 0.000 a	2.80 ± 0.01 e	10 ± 6 d	0 ± 0 b
0	1	0.5	0.25	15 ± 10 a	1.67 ± 0.08 b	5.63 ± 0.25 b	0.084 ± 0.002 a	0.017 ± 0.000 b	5.01 ± 0.00 c	35 ± 10 c	25 ± 25 ab

0	2	0.5	0.25	5 ± 5 ab	1.00 ± 0.00 c	5.10 ± 0.00 c	0.062 ± 0.000 b	0.014 ± 0.000 c	4.43 ± 0.00 d	60 ± 14 b	50 ± 29 a
0.5	0.5	0.5	0.25	0 ± 0 b	0.00 ± 0.00 d	0.00 ± 0.00 f	0.000 ± 0.000 e	0.000 ± 0.000 f	-	80 ± 8 ab	0 ± 0 b
p-values (one-way ANOVA)				0.049*	0.000***	0.000***	0.000***	0.000***	0.000***	0.000***	0.047*

Means ± S.E. with different letters in each column are statistically significantly different from each other based on Tukey's test at a 5% level (p ≤ 0.05). ns p > 0.05, * p ≤ 0.05, *** p ≤ 0.000 [30 days culture, modified MS (x 2FeEDTA) medium + 30 g L^{-1} sucrose + 6 g L^{-1} Plant Agar, pH 5.8].

Table S3. Effect of different auxin types (IBA, NAA, IAA) and concentrations (0, 0.5, 1, 2 mg L^{-1}) on *in vitro* rooting parameters of *Citrus* x *limon* var. *limon* (L.) Burm. f. x *C. latifolia* var. *latifolia*

Treatments (mg L^{-1})	Rooting (%)	Root number/ rooted explant	Root length (cm)	Root FW (g)	Root DW (g)	Root FW/DW ratio
Control	10 ± 10 cd	2.00 ± 0.00 b	3.03 ± 0.16 b	0.038 ± 0.002 de	0.008 ± 0.000 d	5.04 ± 0.01 e
0.5 IBA	0 ± 0 d	0.00 ± 0.00 d	0.00 ± 0.00 f	0.000 ± 0.000 f	0.000 ± 0.000 g	0.00 ± 0.00 f
1 IBA	10 ± 10 cd	2.50 ± 0.04 b	1.28 ± 0.01 e	0.031 ± 0.001 de	0.006 ± 0.000 de	5.17 ± 0.05 de
2 IBA	20 ± 14 c	3.25 ± 0.13 a	1.73 ± 0.06 cd	0.078 ± 0.004 c	0.013 ± 0.001 c	5.78 ± 0.21 cd
0.5 NAA	25 ± 5 c	3.20 ± 0.17 a	2.07 ± 0.14 c	0.095 ± 0.007 bc	0.018 ± 0.001 ab	5.54 ± 0.12 d
1 NAA	80 ± 8 b	3.75 ± 0.41 a	2.08 ± 0.15 c	0.134 ± 0.018 a	0.021 ± 0.003 a	6.41 ± 0.22 b
2 NAA	95 ± 5 a	3.37 ± 0.51 a	1.52 ± 0.23 de	0.106 ± 0.021 b	0.017 ± 0.001 b	6.59 ± 0.45 ab
0.5 IAA	20 ± 8 c	1.25 ± 0.04 c	1.94 ± 0.09 c	0.032 ± 0.002 de	0.004 ± 0.000 ef	5.30 ± 0.20 d
1 IAA	5 ± 5 d	1.00 ± 0.00 c	1.50 ± 0.00 de	0.014 ± 0.000 ef	0.002 ± 0.000 fg	7.00 ± 0.00 a
2 IAA	5 ± 5 d	1.00 ± 0.00 c	3.90 ± 0.00 a	0.041 ± 0.000 d	0.007 ± 0.001 de	5.86 ± 0.00 c
2-way ANOVA/General Linear Model						
Auxin type (A)	0.000***	0.000***	0.000***	0.000***	0.000***	0.000***
Auxin concentration (B)	0.000***	0.000***	0.000***	0.000***	0.000***	0.000***
(A)*(B)	0.000***	0.000***	0.000***	0.000***	0.000***	0.000***

Means ± standard errors (S.E.) with different letters in each column are statistically significantly different from each other based on Tukey's test at a 5% level (p ≤ 0.05). *** p ≤ 0.000 [30 days culture, modified MS (x 2FeEDTA) medium + 30 g L^{-1} sucrose + 6 g L^{-1} Plant Agar, pH 5.8].

Table S4. Effect of different auxin types (IBA, NAA, IAA) and concentrations (0, 0.5, 1, 2 mg L^{-1}) on *in vitro* vegetative growth parameters of *Citrus* x *limon* var. *limon* (L.) Burm. f. x *C. latifolia* var. *latifolia*

Treatments (mg L^{-1})	Shoot height (cm)	Shoot FW (g)	Shoot DW (g)	Shoot FW/DW
Control	1.45 ± 0.08 e	0.097 ± 0.009 cd	0.026 ± 0.002 c	3.67 ± 0.06 b
0.5 IBA	2.03 ± 0.10 a	0.117 ± 0.009 abcd	0.033 ± 0.003 abc	3.53 ± 0.03 c
1 IBA	1.47 ± 0.08 de	0.093 ± 0.006 d	0.028 ± 0.002 c	3.33 ± 0.04 cd
2 IBA	1.71 ± 0.09 cd	0.133 ± 0.009 ab	0.036 ± 0.003 ab	3.66 ± 0.03 bc
0.5 NAA	1.52 ± 0.08 de	0.125 ± 0.012 abc	0.037 ± 0.004 ab	3.40 ± 0.04 cd
1 NAA	1.54 ± 0.08 de	0.143 ± 0.012 a	0.038 ± 0.003 a	3.75 ± 0.03 b
2 NAA	1.53 ± 0.08 de	0.115 ± 0.012 abcd	0.029 ± 0.003 bc	3.96 ± 0.05 a
0.5 IAA	1.78 ± 0.09 bc	0.105 ± 0.008 bcd	0.032 ± 0.003 abc	3.30 ± 0.04 d
1 IAA	1.99 ± 0.09 ab	0.128 ± 0.007 ab	0.037 ± 0.002 a	3.43 ± 0.03 cd
2 IAA	1.91 ± 0.07 abc	0.120 ± 0.007 abcd	0.033 ± 0.002 abc	3.59 ± 0.04 bc

2-way ANOVA/ General Linear Model				
Auxin type (A)	0.000***	0.285 ns	0.637 ns	0.000***
Auxin concentration (B)	0.000***	0.003**	0.001**	0.000***
(A)*(B)	0.000***	0.013*	0.033*	0.000***

Means ± S.E. with different letters in each column are statistically significantly different from each other based on Tukey's test at a 5% level ($p \leq 0.05$). ns $p > 0.05$, *$p \leq 0.05$, **$p \leq 0.01$, ***$p \leq 0.000$ [30 days culture, modified MS (x 2FeEDTA) medium + 30 g L^{-1} sucrose + 6 g L^{-1} Plant Agar, pH 5.8].
